# Incessant Ventricular Fibrillation in a Patient with a Left Ventricular Assist Device and an Implantable Cardioverter-Defibrillator: A Case Report

**DOI:** 10.3390/reports9020117

**Published:** 2026-04-10

**Authors:** Alwin B. P. Noordman, Michiel Rienstra, Alexander H. Maass

**Affiliations:** Department of Cardiology, Heart Center, University Medical Center Groningen, University of Groningen, 9713 GZ Groningen, The Netherlands; a.b.p.noordman@umcg.nl (A.B.P.N.); m.rienstra@umcg.nl (M.R.)

**Keywords:** ventricular fibrillation, implantable cardioverter-defibrillator, unnecessary ICD shocks, left ventricular assist device, case report

## Abstract

**Background and Clinical Significance:** Patients with a left ventricular assist device (LVAD) are at risk of ventricular arrhythmias, which are generally hemodynamically tolerated if they occur. In such cases, patients may experience painful implantable cardioverter-defibrillator (ICD) shocks. **Case Presentation:** A 71-year-old patient with a history of dilated cardiomyopathy caused by a phospholamban (PLN) gain-of-function mutation, with a primary prevention ICD and an LVAD, presented with multiple ICD shocks which she experienced as painful and traumatic. She was found to have ongoing ventricular fibrillation with apparent hemodynamic stability. Conversion to sinus rhythm was achieved through intravenous administration of antiarrhythmic drugs followed by external defibrillation using stacked shocks. Due to the traumatic nature of the shocks, the shock function of the ICD was turned off. Nearly two months later, the patient presented for a second time and was again found to have ventricular fibrillation which had been present for at least six weeks. Conversion to sinus rhythm was unsuccessful and the patient was discharged to her home with an advanced care plan and her LVAD was deactivated. The patient died two months later. **Conclusions:** Patients with an LVAD can remain hemodynamically stable for prolonged periods of time during ventricular arrhythmias. ICD shocks are therefore mostly experienced as painful and even traumatic. Therefore, the routine use of ICD shock therapy in patients with an LVAD should be reconsidered. Adjustment of ICD programming to higher rates and longer detection may be warranted. Further investigation is warranted regarding a switch to devices with an alarm function rather than therapies for tachyarrhythmias.

## 1. Introduction and Clinical Significance

A left ventricular assist device (LVAD) is part of advanced heart failure treatment, implanted either as destination therapy or as a bridge to heart transplantation. Patients with an LVAD are known to be at risk for ventricular arrhythmias, with the ASSIST-ICD study demonstrating that 27% of patients experience late ventricular arrhythmias [1], and another study reporting incidences ranging between 20% and 60% [2]. Ventricular arrhythmias have been shown to be associated with an increased risk of mortality in patients with an LVAD [3]. However, when ventricular arrhythmias occur in these patients, they mostly remain hemodynamically stable due to the mechanical support provided by the LVAD. For instance, a previous study described a cohort of 19 such patients, who remained awake in the presence of ventricular fibrillation (VF) or ventricular tachycardia (VT) [4]. The time in VF is usually not more than a few minutes, however, although there have been patients with an LVAD who have been reported to be in VF for many months [5,6]. When patients with an LVAD additionally have an implantable cardioverter-defibrillator (ICD), they are at risk of experiencing painful shocks as a result of such tachyarrhythmias, which have been found to occur in a substantial number of patients [7].

Though previous case reports have described the occurrence of VF in patients with an LVAD, there has been a lack of reports describing the interaction between the LVAD and ICDs. The impact of shocks, more specifically unnecessary shocks, occurring in this patient population is a topic underrepresented in the existing literature and deserves further exploration.

In this case report, we describe a patient with an LVAD who had VF for at least six weeks and who experienced painful, traumatic ICD shocks.

## 2. Case Presentation

**Figure 1:**
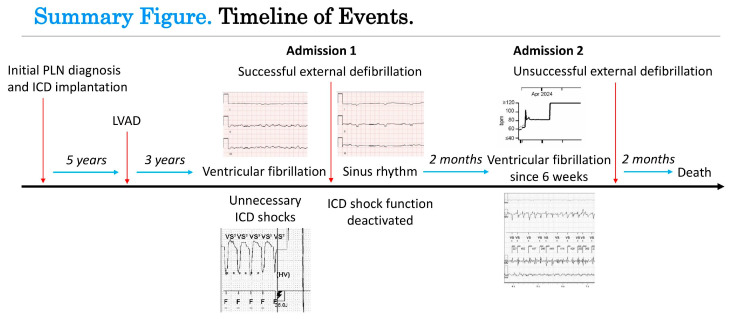
Summary figure displaying a timeline of events for the case presented, with two presentations to the hospital. ICD, implantable cardioverter-defibrillator; LVAD, left ventricular assist device; PLN, phospholamban.

**Figure 2:**
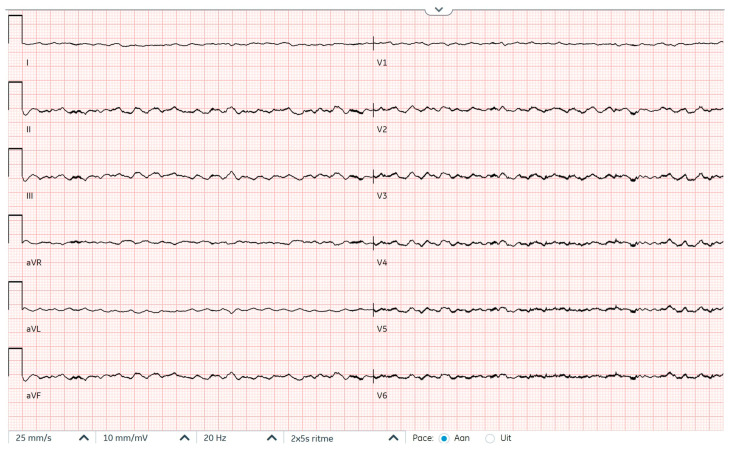
Electrocardiogram at first presentation demonstrating ventricular fibrillation after filtering out LVAD signals.

**Figure 3:**
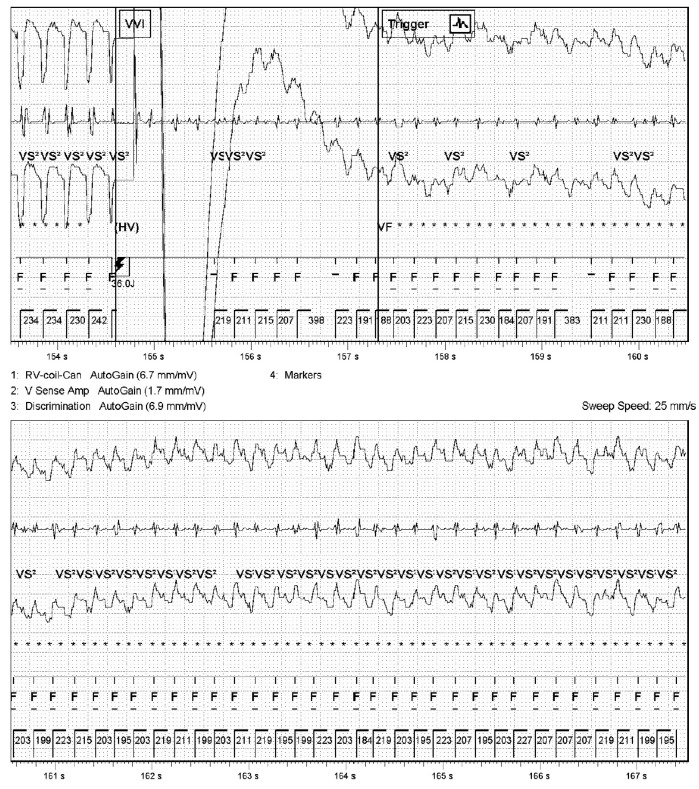
Electrogram as stored by the implantable cardioverter-defibrillator showing the delivery of an appropriate shock for VT, which then degenerates into ventricular fibrillation. ICD settings were as follows: VT monitoring zone 150/min, 30 intervals to detect; VF zone 200/min, 60 intervals to detect with ATP 1x, 36 J shock, 40 J shock, 40 J shock 4x, biphasic waveform; sensing value 6.2 mV. ATP, antitachycardia pacing; VF, ventricular fibrillation; VT, ventricular tachycardia.

**Figure 4:**
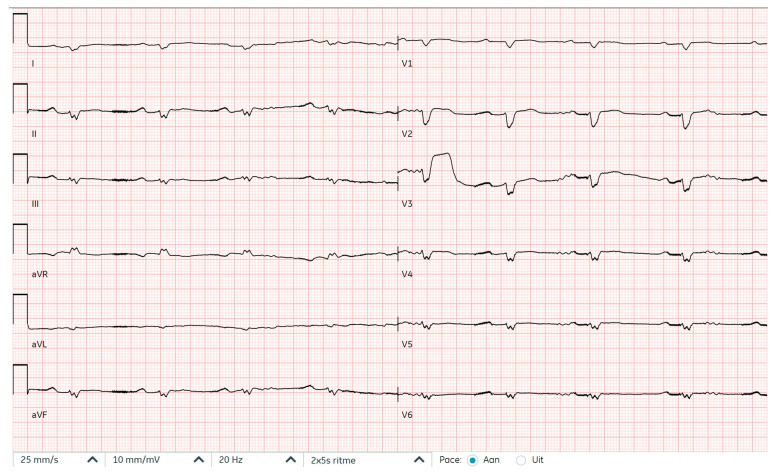
Electrocardiogram after conversion to sinus rhythm after filtering out LVAD signals.

In this case report we describe two presentations to the hospital of a 71-year-old female patient with a history of dilated cardiomyopathy caused by a phospholamban (PLN) gain-of-function mutation and a VVI-ICD implanted for primary prevention of sudden cardiac death (Figure 1). Years after the ICD implantation, an LVAD HeartMate 3 was implanted as destination therapy because of end-stage heart failure and repetitive episodes of ventricular tachyarrhythmias. This was complicated by a driveline infection treated with antibiotics. She was not considered a suitable candidate for heart transplantation because of her age and renal dysfunction. Several years later, a transfemoral transcatheter aortic valve implantation was performed for severe aortic insufficiency. Two months later, at the time of the first presentation, the patient reported experiencing a flu-like feeling for one day. She then experienced multiple ICD shocks which she felt to be very painful and which took her by great surprise. For this reason, she called the ambulance, who transported her to the emergency department with ongoing VF with apparent hemodynamic stability (blood pressure 70/50 mmHg, maximal EMV score, awake and mentally lucid) (Figure 2). ICD interrogation demonstrated VF for which a multitude of unsuccessful ICD shocks were delivered. The ICD electrograms showed that the episode started with VT, which accelerated and changed to become VF after a shock (Figure 3). At no point during VF had the patient lost consciousness (LVAD settings two weeks prior to first presentation: pump flow 3.8 L/min, pump speed 5000 RPM, pump power 3.4 W; at discharge of first presentation: pump flow 3.6 L/min, pump speed 4800 RPM, pump power 3 W). Echocardiography showed a dilated right ventricle and an underfilled left ventricle. The patient had already received amiodarone intravenously in the ambulance. In the emergency department, external defibrillation was performed but was not successful. Procainamide and metoprolol were then administered intravenously, after which external defibrillation using stacked shocks was performed, which proved successful (Figure 4). The incessant VF was attributed to right heart failure and progression of her underlying cardiomyopathy. The patient was admitted for approximately one month, during which the patient was treated for right heart failure and with amiodarone. Before admission, the medication used by the patient included acenocoumarol, amiodarone, carbasalate calcium, bisoprolol, bumetanide, pantoprazole, sacubitril–valsartan, spironolactone. The maintenance dosage of amiodarone and bisoprolol was increased (amiodarone from 200 mg once daily to 300 mg once daily and bisoprolol from 1.25 mg once daily to 5 mg once daily), while sacubitril–valsartan was terminated during admission. The ICD shocks that the patient had experienced during full consciousness had been very traumatic, such that she kept thinking about them and relived them, especially at night. The shocks thus caused direct harm and substantially reduced quality of life. In fact, they were so traumatic that the shock function of the ICD was turned off definitively as per the patient’s request. The patient was informed of the potential consequences of such a decision. Ultimately, the decision to turn off the shock function of the ICD was made in consultation with the patient through shared decision-making to ensure a well-informed decision. Thus, patient preferences guided management. Because of the right ventricular failure, maintenance treatment with sildenafil was initiated to reduce pulmonary vascular resistance, which was stopped about two months later due to bothersome side effects.

**Figure 5:**
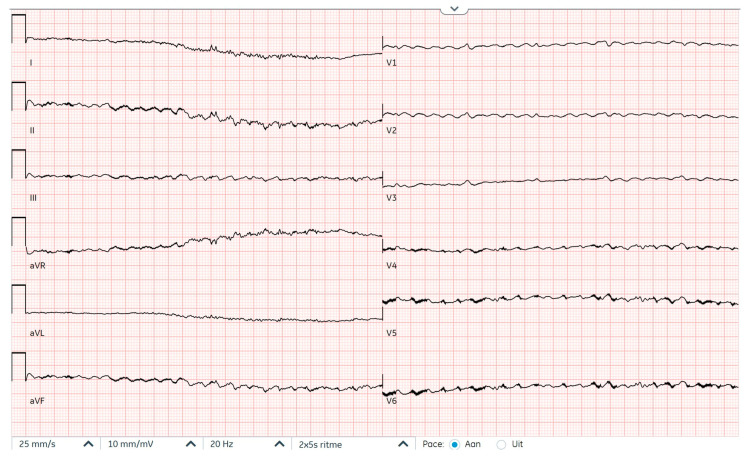
Electrocardiogram at second presentation demonstrating ventricular fibrillation after filtering out LVAD signals.

**Figure 6:**
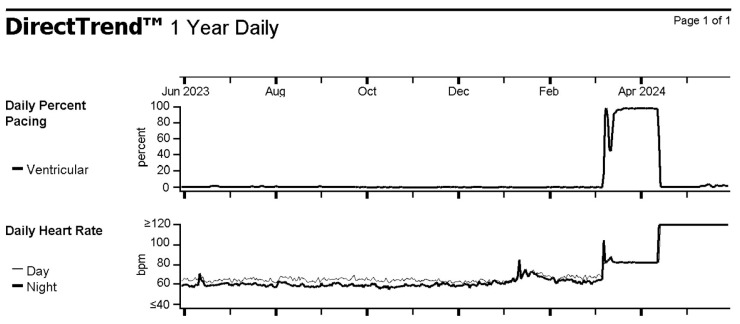
Interrogation of the implantable cardioverter-defibrillator showing a heart rate faster than 120 beats per minute for six weeks corresponding to ventricular fibrillation.

**Figure 7:**
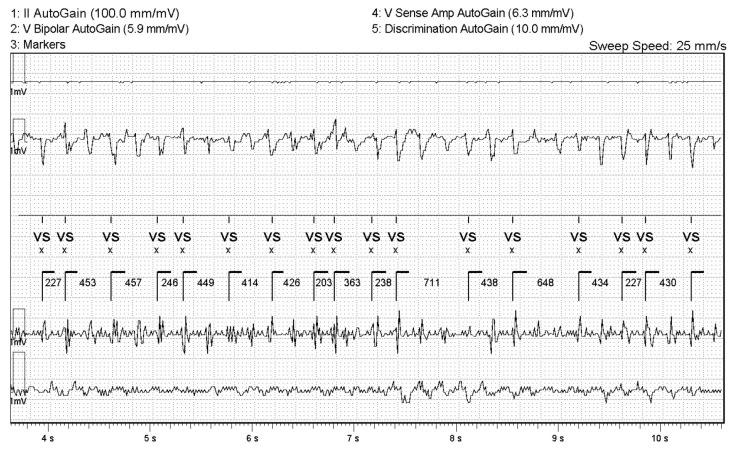
Electrogram at the time of interrogation of the implantable cardioverter-defibrillator showing ventricular fibrillation.

Nearly two months later, the patient presented with a health situation that had worsened over several months. She had dyspnea during exercise and felt that her heart and her body were failing her. She additionally described having lost consciousness several times when going to the bathroom at home. At presentation in the emergency department she was conscious and in a clear state of mind. Electrocardiography showed VF (Figure 5). ICD interrogation showed that the patient had been in VF for 6 weeks already (Figure 6 and Figure 7). External defibrillation under conscious sedation was performed and resulted in restoration of a normal heart rhythm for a very brief amount of time, but very soon VF started again. Since VF recurred so quickly after defibrillation and had been present for so long already, with no clear treatable triggering factor except for progression of her underlying cardiac disease, while the patient had been admitted with the same problem quite recently, it was decided that there were no long-term treatment options. Furthermore, all of this was placed in the context of her underlying end-stage heart failure caused by a PLN mutation in the presence of an LVAD as destination therapy. Based on the aforementioned information, VF was accepted as terminal rhythm. Amiodarone could therefore have been discontinued. The patient was aware that the end of her life had come and that she was going to die relatively soon. The poor prognosis was explained to the patient and her family, as well as the fact that there were no long-term treatment options. Thus, clinical decision-making was conducted in consultation with the patient and her family, while conversations were clinically documented. She was discharged to her home with an advanced care plan and her general practitioner would take over her care, including ultimately the deactivation of the LVAD and palliative sedation if needed. All of this was discussed and decided upon in consultation with the patient through shared decision-making, while taking into consideration ethical considerations and patient preferences. A week later, the patient was in a palliative care trajectory. She was mostly confined to bed and said she felt weak. Two months later, she died.

To conclude, this case report describes a case of persistent VF as a result of progressive cardiac pathology, and the occurrence of harmful ICD shocks in a patient with an LVAD for end-stage heart failure due to a PLN mutation.

## 3. Discussion

This case report adds to the reports of patients with LVADs who experience VF while remaining fully conscious. Repetitively occurring ventricular arrhythmias as in the case of electrical storm are not extremely rare in patients with an LVAD, with the incidence of electrical storm being approximately 9% [8]. However, most case reports to date have reported a shorter duration of VF than was the case for the patient described herein, who was in VF for at least six weeks. More importantly, however, this case shows the harmful nature of ICD shocks in patients with an LVAD, whose circulation is generally maintained in the presence of ventricular tachyarrhythmias through mechanical support provided by the LVAD. Such shocks may therefore be considered unnecessary. Our case thus calls into question the standard use of ICD shock therapy in LVAD patients.

ICD shocks may be experienced as painful and result in a reduction in quality of life [9], as was the case in our patient. The delivered shocks were not only ineffective but were also harmful for the patient, because they caused significant physical and psychological distress. For this reason, the patient wished for the ICD shock function to be turned off. In general, it may therefore be warranted to adjust ICD programming to higher rates and longer detection in patients with an LVAD so that they are less likely to experience such unnecessary shocks [10]. A previous study found that ICD programming with maximal detection duration while maximizing antitachycardia pacing before shocks likely resulted in a decrease in unnecessary ICD therapies, while the corresponding episodes of ventricular arrhythmias were not associated with symptoms indicating hemodynamic instability [11]. In our case, if ICD settings had been more conservative from the outset, this might have prevented the occurrence of unnecessary shocks, also considering the fact that VF arose after the occurrence of a shock for VT. However, this remains speculation and cannot be known. In fact, the role of the ICD in patients with an LVAD has been questioned, considering the findings of a previous study that an ICD implanted either prior to or after an LVAD implantation was not found to result in a survival benefit [12]. Our case highlights the potential burden of ICD shocks in LVAD-supported patients and suggests that individualized ICD programming strategies may be warranted. However, larger studies are required before changes to current clinical practice can be recommended. Aside from adjustments in ICD programming, catheter ablation is another management strategy that deserves mentioning, although it was not employed in the case presented. It has previously been shown in patients with an LVAD that VT ablation is associated with a reduction in therapies delivered by the ICD, although the rate of recurrence of VT was high [13]. Another management strategy concerns thoracoscopic sympathectomy, which may offer patients freedom from VTs in the case of otherwise unmanageable and recurring VTs [14].

Even if patients with an LVAD can survive for prolonged periods of time in VF, secondary consequences may result from this arrhythmia, including the development of an intracardiac thrombus which may embolize, as well as right ventricular failure and eventually organ failure. During the first presentation to the emergency department of our patient, echocardiography showed a dilated right ventricle and an underfilled left ventricle. This indicates that there already was a right ventricular volume overload. Because of these deleterious secondary consequences, it is undesirable for VF to exist for prolonged periods, therefore warranting early attempts at restoration to sinus rhythm. For this, an ICD is not necessary per se. Instead, early external defibrillation may be performed while the patient is sedated.

ICDs may not prevent sudden cardiac death or all-cause mortality in LVAD recipients [15]. Only the monitoring function may be of value in those who already have an ICD prior to LVAD implantation. Therefore, patients that have already received an ICD should have their device programmed to high rates and long detection times and even the deactivation of ICD therapies or extraction of hardware during LVAD placement could be considered in selected patients. Such measures have, however, been insufficiently studied, and it is important to mention that such decisions are to be made on an individual basis together with the patient [16]. We previously proposed the use of patient confirmation through a novel mobile application that alerts patients of an impending ICD shock and allows them to avert unnecessary and inappropriate shocks [17]. LVAD patients in particular are likely to benefit from withholding ICD therapies and are thus likely to benefit from such an application. In patients with no ICD prior to the implantation of an LVAD and in whom continuous monitoring is warranted, an implantable loop recorder may be a good option. Consumer-grade wrist-worn devices, such as smartwatches, may potentially aid in the detection of ventricular arrhythmias, although this deserves further elucidation in future studies [18]. Implantable or wearable devices alarming in the case of sustained ventricular arrhythmias with or without connecting with emergency medical services may be investigated in future studies. This would have the advantage of averting unnecessary ICD shocks while still allowing for the early detection of ventricular tachyarrhythmias. The LVAD itself may potentially be used for this purpose as well. In any case, it is important that an alarm function is incorporated in the applied monitoring strategy. The above-mentioned information makes it clear that there is a need for standardized, LVAD-specific ICD and arrhythmia management protocols informed by findings from studies. However, for this, further prospective studies are needed, specifically regarding the optimization of ICD programming in the LVAD population as well as quality of life studies incorporating patient experiences of ICD shocks.

The novelty of the present case report lies in its demonstration of the unintended and harmful consequences of appropriate yet unnecessary ICD shocks in an LVAD patient who had an extraordinarily long duration of conscious VF, ultimately leading to ICD shock deactivation through shared decision-making. This is a topic underrepresented in the existing literature. By presenting potential alternative management strategies that emphasize the detection of ventricular arrhythmias over their treatment through unnecessary ICD therapies, such as adjustments in ICD programming and alternative detection strategies including a yet to be developed novel mobile application, we provide insights that help to improve the quality of life of patients. Even though not all of the discussed management strategies were employed at the time in the clinical course of the patient, our case does present a great need for change. Other aspects unique to our case report include the presence of a PLN cardiomyopathy, a period of time of VF lasting longer than usually described in the existing literature, and the fact that our case report features the interaction of ICD and LVADs with resultant impactful, unnecessary ICD shocks demonstrating current needs and challenges in this patient population and underscoring the need for a shift in the clinical paradigm of patient care. Finally, by incorporating a focus on patient experience regarding the series of events as well as the care of the patient, we were able to demonstrate the harmful nature of such shocks.

## 4. Conclusions

In conclusion, patients with an LVAD can remain hemodynamically stable during ventricular arrhythmias for a prolonged period of time. Consequently, ICD shocks are mostly experienced as painful and even traumatic by the fully conscious patient. Adjustment of ICD programming to higher rates and longer detection may be warranted. A switch to devices with an alarm function instead of therapies for tachyarrhythmias warrants investigation.

## Data Availability

The original data presented in the study are included in the article, further inquiries can be directed to the corresponding author.
